# Decreased Fixation Stability of the Preferred Retinal Location in Juvenile Macular Degeneration

**DOI:** 10.1371/journal.pone.0100171

**Published:** 2014-06-17

**Authors:** Richard A. I. Bethlehem, Serge O. Dumoulin, Edwin S. Dalmaijer, Miranda Smit, Tos T. J. M. Berendschot, Tanja C. W. Nijboer, Stefan Van der Stigchel

**Affiliations:** 1 Experimental Psychology, Helmholtz Institute, Utrecht University, Utrecht, The Netherlands; 2 Rudolf Magnus Institute of Neuroscience and Centre of Excellence for Rehabilitation Medicine, University Medical Centre Utrecht and Rehabilitation Centre De Hoogstraat, Utrecht, The Netherlands; 3 University Eye Clinic Maastricht, Maastricht, The Netherlands; 4 Autism Research Centre, Department of Psychiatry, University of Cambridge, Cambridge, United Kingdom; National Institute of Mental Health, United States of America

## Abstract

Macular degeneration is the main cause for diminished visual acuity in the elderly. The juvenile form of macular degeneration has equally detrimental consequences on foveal vision. To compensate for loss of foveal vision most patients with macular degeneration adopt an eccentric preferred retinal location that takes over tasks normally performed by the healthy fovea. It is unclear however, whether the preferred retinal locus also develops properties typical for foveal vision. Here, we investigated whether the fixation characteristics of the preferred retinal locus resemble those of the healthy fovea. For this purpose, we used the fixation-offset paradigm and tracked eye-position using a high spatial and temporal resolution infrared eye-tracker. The fixation-offset paradigm measures release from fixation under different fixation conditions and has been shown useful to distinguish between foveal and non-foveal fixation. We measured eye-movements in nine healthy age-matched controls and five patients with juvenile macular degeneration. In addition, we performed a simulation with the same task in a group of five healthy controls. Our results show that the preferred retinal locus does not adopt a foveal type of fixation but instead drifts further away from its original fixation and has overall increased fixation instability. Furthermore, the fixation instability is most pronounced in low frequency eye-movements representing a slow drift from fixation. We argue that the increased fixation instability cannot be attributed to fixation under an unnatural angle. Instead, diminished visual acuity in the periphery causes reduced oculomotor control and results in increased fixation instability.

## Introduction

Juvenile macular degeneration (JMD) affects approximately 1 in 10.000 individuals [1]. Most often it is caused by mutations in the ABCA4 gene, which transcribes a large retina-specific protein, leading to Stargardt disease [2], [3], [4]. As a result patients commonly develop a central scotoma that involves the fovea. The resulting loss of foveal vision has a severe impact on patients visual acuity. Early research has shown that this can be accompanied by a shift in the oculomotor reference from the fovea to a nonfoveal locus [5]. Subsequently patients suffering from macular degeneration often adopt one or multiple preferred retinal loci (PRL) that can serve as a ‘pseudo-fovea’ [6], [7]. This PRL is an eccentric location on the retina that is used for fixation in favor of the fovea. Crossland et al. [8] have shown that this PRL can develop within six months of visual loss onset and further research shows that the PRL location can remain relatively stable in people with age-related macular degeneration [9]. Additionally, strategies for developing a PRL appear to be slightly different for different causes of macular degeneration [10], with Stargardts disease being the more variable one. Apart from the obvious poorer resolution of the visual retina in the periphery, fixation with the PRL in patients with MD (Throughout this manuscript we use JMD to refer to juvenile macular degeneration, MD to refer to non-specific macular degeneration and AMD to refer to age-related macular degeneration) also tends to be unstable [11], [12]. In healthy individuals fixation instability can be beneficial in the central retina because of a low tolerance for image motion [13], [14]. As resolution decreases with higher velocity eye movements [14] fixation instability recovers some of this loss [13]. During stable fixation peripheral vision also tends to decrease (Troxler fading) and unstable fixation partially recovers this fading. As Deruaz et al point out this has led to the suggestion that increased fixation instability for people that use peripheral fixation (such as people with MD) might be equally beneficial [15]. However, Macedo et al. have showed that this is not necessarily the case for patients with MD using peripheral vision [16]. Specifically, Macedo and colleagues [16] showed that fixation instability does not reduce crowded or non-crowded visual acuity. Thus, in standard reading or acuity test this instability does not produce any net benefit for people with MD. At the same time, paradigms utilizing visual acuity types of tests might not be the best predictors in determining fixation patterns and rehabilitation outcomes in patients with macular degeneration [17].

Most studies to date have not explicitly focused on the juvenile form of macular degeneration but on age-related macular degeneration (AMD) [10], [17], [18], [19], [20], [21], [22], [23], [24]. AMD is the most common form and is considered to be the main cause of diminished visual acuity in the elderly [25]. One study has shown that in AMD training can significantly improve fixation stability [24]. It has also been shown that fixation stability can be strongly correlated with PRL eccentricity [23]. In addition, Tarita-Nistor et al. [21] have shown that patients with AMD generally have good ocular motor coordination and fixation control, but that this disappears when one eye is covered. In addition, it has been shown that fixation characteristics may differ between monocular and binocular viewing in patients with AMD when asked to fixate for relatively long time periods [26]. It is possible that the relative late onset of AMD compared to JMD allows for some sustaining of oculomotor control and that this is better during binocular viewing [21]. In the present study we will focus specifically on the ability to keep fixation steady for short periods during binocular viewing by individuals with JMD.

Most studies which have investigated fixation characteristics of patients with macular degeneration have used a microperimeter [18], [19], [23], [24], [27] to assess fixation stability of the PRL. This measure generally has a low temporal resolution (<25 Hz) and as a result partial saccades (start or end points) are often difficult to take into account, let alone remove from the data. Additionally, long fixation periods are often used (exceeding 10s) which might make it harder for participants to stay focused. In contrast, short fixation phases might give a more accurate account of fixation characteristics under ecologically important and valid conditions such as during reading, visual search, visual scene processing or even typing. These types of fixation phases generally fall well within a 150 to 450 ms. time-scale [28]. In addition, precisely these types of viewing conditions have been shown to be impaired in MD [11], [29]. Infrared eye-trackers allow short but detailed recording at this high temporal resolution as well as detection of trials containing saccades that can subsequently be removed. Infrared eye-trackers have already shown high test-retest reliability and have been proven useful in the assessment of fixation characteristics when compared to standard Scanning Laser Ophthalmoscope (SLO) measurements [30]. Although Crossland and Rubin [30] show that the eye-tracker methodology tends to overestimate the fixation instability, they also argue that this overestimation might be caused by small compensatory eye-movements. These are a result of the fact that participants’ heads were completely unrestrained during testing. In addition, because infrared eye-trackers can record at high temporal resolutions, the need for long fixation periods becomes unnecessary and paves the way for more ecologically valid ways of assessing fixation in patients with MD. Furthermore, they allow for more fine-grained analyses of eye-tracking data such as power spectral densities with high temporal resolution that can further elucidate underlying fixation characteristics [31].

In the present study we aim to investigate the fixation characteristics in a group of patients with JMD that have stable PRL’s during a paradigm that can potentially distinguish between foveal and peripheral types of fixation using an infrared eye-tracker. To this end we adopted a fixation offset paradigm [32]. This paradigm includes a short fixation phase that covers the range of fixation times reported during various different types of viewing [28] without explicitly restraining the fixation characteristics by a specific cue. In this paradigm participants do not focus explicitly on a fixation cross but instead are shown fixation anchors at a distance of either 1° degree or 3°. Machado and Rafal [32] have shown that in healthy controls the 1° condition represents a foveal specific type of fixation whereas the 3° does not. In the 1° condition, introducing a gap between target onset and fixation offset results in a decrease in saccade latencies which does not occur in the 3° condition [33]. Fendrich et al. [33] argue that the 1° anchor falls within foveal fixation and the gap-effect thus only occurs for foveal fixation. In a previous study we have shown that a central scotoma combined with peripheral viewing impairs search efficiency and that these results can be explained without the necessity of reorganisation in the visual system [29]. Since the JMD group will use their periphery for both the foveal as well as the peripheral fixation anchor conditions the difference in fixation stability should be minimal. For the control group we would expect a difference for foveal (1 degree condition) versus non-foveal (3 degree condition) fixation, whereby the non-foveal fixation may resemble the general fixation of the patient group more closely.

We hypothesize that patients with JMD will have greater overall fixation instability due the use of peripheral viewing when compared to controls. Based on our previous study [29] using a visual search paradigm we do not expect that the PRL will have adopted foveal fixation properties and thus we do not expect an effect of fixation anchor-size. In contrast, we expect healthy controls to have an overall more stable fixation pattern that is strongest in the foveal (1 degree) fixation condition. To assess this instability, we will first look at the number of intrusive saccades, defined as a saccade during a moment in the task where stable fixation is required. Second, as a measure of fixation instability we use a bivariate contour ellipse area (BCEA) [23], [30], [34], [35] and the overall displacement during the course of the fixation period. Finally, to further investigate the nature of fixation in JMD we will analyze the power spectral densities [31]. To ensure that any fixation instability is not explained by an ‘unnatural’ position of the eye during eccentric viewing we also tested a simulated PRL version of this paradigm in healthy controls.

## Methods

### Ethics Statement

The ethical institutional review board of the University Medical Centre Utrecht approved this study, and all subjects gave written informed consent prior to participation. All study procedures have been conducted according to the principles expressed in the Declaration of Helsinki.

### Participants

For this study, we recruited 10 patients with juvenile MD. Additionally, 10 healthy age-matched controls participated in the same paradigm and another 6 healthy controls were recruited for participation in a simulation version of this paradigm. The JMD participants had an official diagnosis of JMD (assessed by their own physician and confirmed by means of a questionnaire) and had no history of neurological and/or psychiatrical disorders or substance abuse. Controls had normal or corrected to normal acuity and had no history of neurological and/or psychiatrical disorders or substance abuse either. All participants received 20 euro and travel expenses for their participation.

### Procedure, Stimuli and Design

Clinical characteristics of the JMD patients were verbally interrogated by means of a questionnaire, see Table 1. After filling out the questionnaire, the experimental procedure started. All measurements were conducted in a sound-attenuated, dimly lit room. Eye movements were recorded by an Eyelink1000 system (SR Research Ltd, Canada), an infrared video-based eye-tracker. The dominant eye, which was verified with a visual alignment task [36], was monitored and analysed in all participants. The non-dominant eye was not occluded during the course of the experiment to allow for naturalistic viewing. Although no research to date has established a clear link between eye dominance as measured by an alignment task and the dominant eye for fixation, this was the least arbitrary way to determine which eye to track. The participants’ heads were stabilized using a chin rest to control for compensatory head movements. We acknowledge that head stabilization may somewhat limit the ecological validity, but it was necessary to make full use of the eye trackers temporal and spatial specificity. The distance between monitor and chin rest was 57 cm. A nine-point grid calibration and subsequent validation procedure was utilized before the start of the experiment.

**Table 1:** Clinical characteristics of individual patients.

Patient	Gender	Official Diagnosis	Age (y)	Age onset (y)
MD1	F	Stargardt	33	23
MD2	F	Stargardt	29	12
MD3	M	X-Chromosal schisis*	48	congenital
MD4	M	Stargardt	47	gradual
MD5	M	Stargardt	23	6
MD6	F	Best’s Disease	38	20

*X-chromosome-linked juvenile retino- schisis.

### Scanning Laser Opthalmology

**Figure 1:**
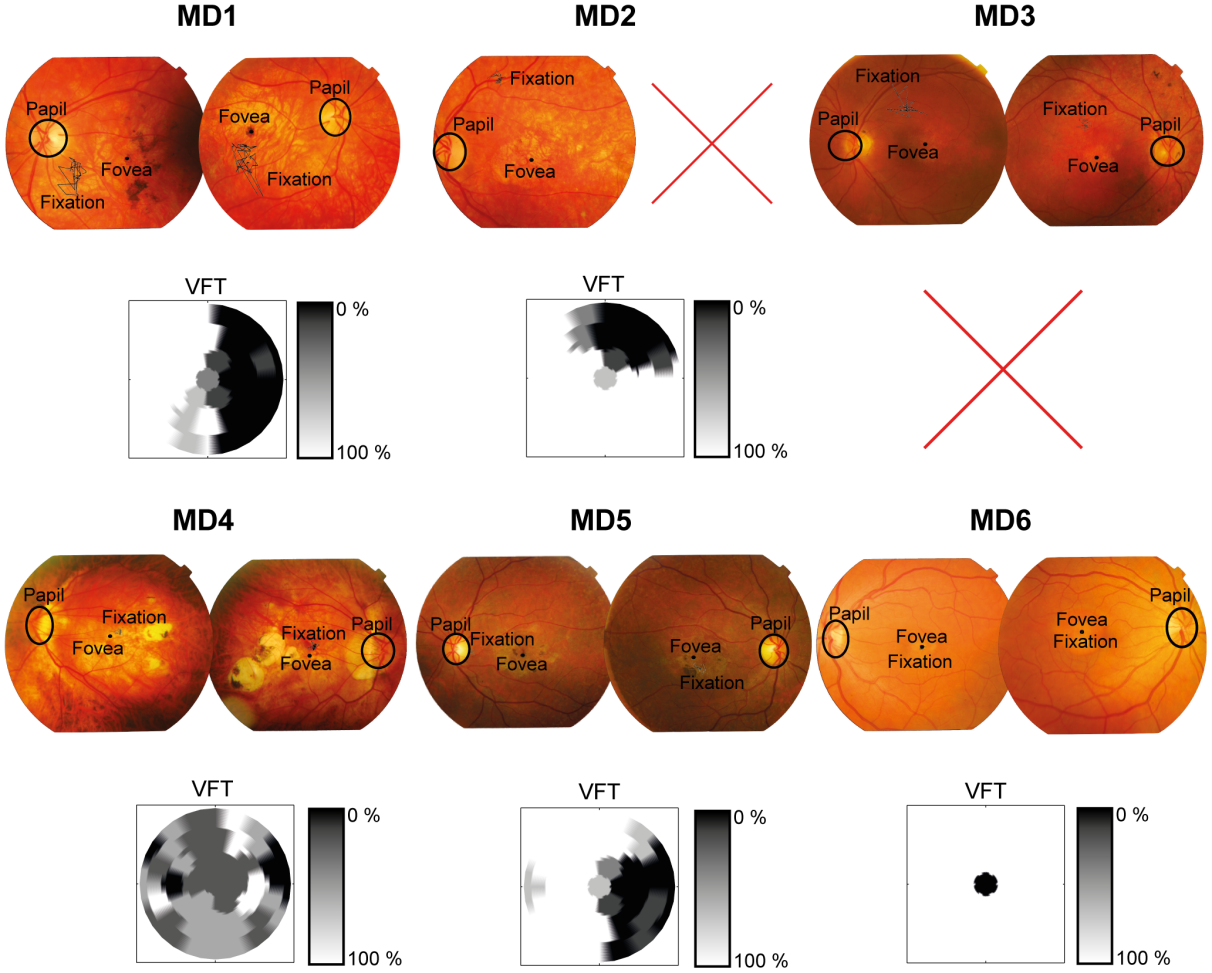
Scanning Laser Opthalmoscope photo’s. SLO photographs of all JMD participants. Participant 6 (lower right) was excluded due to the evident overlap of fixation and fovea. Interpolated visual field task images are shown below each respective SLO image. These show the visual field defect (VFT) for an 18° by 18° degrees visual field. Dark areas represent the point in the visual field where there was a defect, white represent no defect (ranging from 0–100%). Because the VFT measurements are based on binocular viewing and the SLO images are from each eye separately they not always clearly translate to one another [21], [26].

In order to gain information about fixation stability and absolute locus of the PRL, patients were invited for a separate Scanning Laser Ophthalmoscope measurement (SLO) at the University Eye Clinic Maastricht [37]. We used a custom build Scanning Laser Ophthalmoscope [37], [38] to image the fundus and to present the stimulus. The subjects fixated on a red cross that was presented in the SLO. To determine the absolute location of the PRL at the retina and its stability, we acquired 60 SLO images per participant with the use of a frame grabber, having a 1 sec interval in between. Similarly as described in Reinhard et al. [39] we used an SLO that shows the fundus and the fixation cross simultaneously on a video monitor. Further analysis was done also similar as described in the Reinhard et al. paper [39]. We aligned the subsequent images and calculated the PRL and its movement. Images are shown in Figure 1. The fundus photographs show the location of this PRL over time.

### Visual Field Test

In addition, we used a visual field test to confirm the absolute visual field defect. In the visual field test, one target at a time was shown and a fixation cross was used which remained on screen during presentation of the target. The target was a black 1.5° dot and could appear at 33 possible stimulus locations with a background luminance of 52.95 cd/m2. The target was presented for 1500 ms. The 33 locations were organised in five rows; three rows consisted of seven locations and two rows of six locations. The centre-to-centre distance, both within and between rows of each location had a visual angle of 5°. Participants were instructed to remain fixated on the fixation cross and report, using the ‘z’ and ‘/’ keys, whether they had seen a target or not. After their response a confirmation of their choice was presented on screen. Target present trials were mixed with 'catch' trials in which no target was presented. All target locations were presented four times along with 16 catch trials, making for 148 trials in total [29]. The visual field defect has been incorporated in Figure 1. MD case number 6 was excluded based on converging evidence from both the SLO and visual field test that the fixation overlapped with the fovea. For MD 3 there was a technical issue with the visual field data, but the SLO showed clear use of a PRL.

### Fixation Offset Paradigm

**Figure 2:**
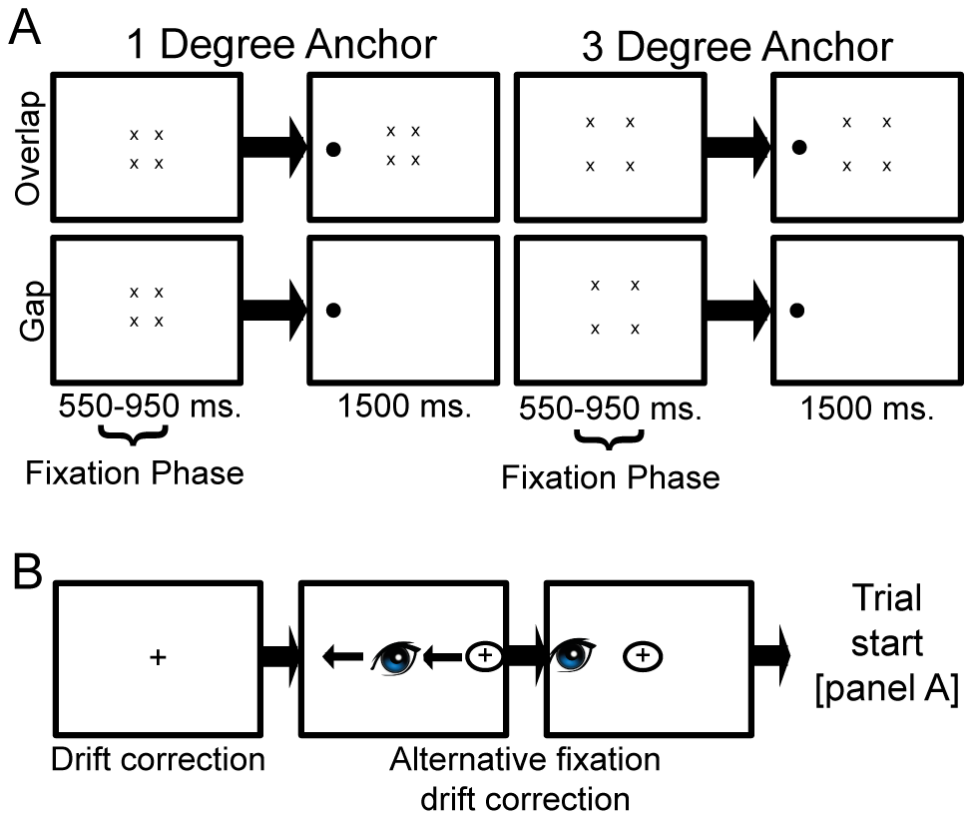
Paradigm overview. Panel A shows a schematic overview of the fixation-offset paradigm as used by Machado & Rafal [32]. After drift correction participants are instructed to keep a steady fixation within the four anchors. As soon as a target appears they are instructed to make an eye-movement to that target as fast as possible. Presently, we focussed on the fixation phase (before target presentation) of this paradigm. Panel B shows the adaptation used in the PRL simulation version of this paradigm. Prior to the normal trial procedure (but after drift correction), participants had to align a gaze-controlled alternate eccentric fixation point over a central fixation cross. This led them to use their peripheral vision to fixate on the central fixation cross before the start of the trial. In panel B the “eyeball” symbol represent the true fixation, the ‘+’ sign represents the central fixation and the circled ‘+’ sign represents the eccentric fixation point that was controlled by the participants eye movement. There was no minimum fixation time during alignment of the eccentric fixation point that participants had to maintain for the trial to start. However, should participants make a saccade directing the eccentric fixation to the central fixation cross and then press the spacebar, the subsequent saccade parser would have detected a saccade at trial start and the trial would have been removed from subsequent analysis.

To test fixation characteristics we used a fixation-offset paradigm [32]. All trials started with a drift check to ensure the calibration was still accurate. Participants were instructed to fixate on an unmarked centre containing four eccentric anchors surrounding the *unmarked* centre (background luminance of 32.07 cd/m2). The unmarked centre served as the fixation point and was located at the centre of the display. The eccentric fixation anchors consisted of four black crosses (0.64°**×**0.64°) and were presented on the corners of an unmarked square. The distance from the crosses to the centre of the screen was either 3° or 1°. After a pseudo-random interval (between 550 and 950 ms.), a black target circle appeared (diameter of 1.43°). See figure 2A for an overview. In the patient group the location of target dots was dependent of the scotomatous area (either left, right, above or below the eccentric fixation anchor) as target locations that fell within the scotoma, as assessed with a visual field test, were removed from the location possibilities. The eccentric anchors were the same as in the control group to minimize potentially biasing the fixation stability by using different fixation anchors. In the control group targets were presented in all four (left, right, above and below the fixation anchor) possible locations. Participants were instructed to fixate at the *unmarked* centre until the target dot appeared, and subsequently were to move their eyes as fast as possible to the target circle. The target display was presented for 1500 ms. Afterwards all objects were removed from the display. The experiment consisted of 240 experimental trials and 24 practice trials.

### Fixation Offset Simulation Paradigm

To investigate whether any difference in fixation might be caused by an unnatural eye-position in the patient group we also conducted a separate simulation in healthy controls. In this adaptation a para-foveal fixation cross at 8° eccentricity is presented at the right side of the true fixation. This eccentric fixation is an offset of the eye-position as measured with the eye-tracker and is thus controlled by participants’ eye-movement. Participants are instructed to move this alternative fixation point over a centrally located fixation cross, hold their fixation steady and press the spacebar. When this alternative fixation was stable within 2° degrees of the central fixation cross the trial started by removal of the central fixation point. See figure 2B for a graphical overview. At the same time as the central fixation cross disappeared, fixation anchors were presented at either 1° or 3° degrees eccentricity from fixation. These were exactly the same as in the original paradigm and were presented for the same pseudorandom interval (550 ms-950 ms). After this a target was presented above, below or to the left of the true fixation and participants were instructed to move the eyes there as fast as possible and press the spacebar once they had done so. Size of the target dots, fixation crosses and fixation anchors as well as all luminance ratios was kept the same as in the original paradigm. This task was programmed using PyGaze [40]. We acknowledge that the simulation group cannot be considered the ideal comparison to the behaviour observed in the MD group. A gaze-contingent eccentric fixation anchor might not be the best reflection of the deficit that people with MD experience and an alternative might have been the use of a gaze-contingent artificial scotoma. However, we chose not to use a gaze-contingent artificial scotoma because this would have provided the healthy participants of the simulation study with a strong cue (namely the border of the artificial scotoma) to be aligned with the eccentric fixation anchors. Furthermore, an artificial gaze-contingent scotoma is still always visible to a healthy control subject and is thus likely to affect the oculomotor programming. Also, healthy subjects might not necessarily deviate attention to a peripheral location when the artificial scotoma is visible. Instead they might simply attend to the borders of the artificial scotoma. We aimed to minimize these effects by using a gaze-contingent eccentric anchor instead.

### Data Analysis

Our main question concerned the fixation behaviour with patients with JMD and a stable PRL in the absence of a clear fixation point. Therefore we focussed our analysis on the pseudo-random fixation phase at the start of each trial during which participants have to keep their fixation steady and within which the anchor points are presented on screen (Figure 2A).

Three main measures were taken during this period. First, we determined the number of saccades during the fixation phase (figure 2A). Thresholds for detecting the onset of a saccadic movement were an acceleration of 8000°/s2 and a velocity of 30°/s. These are the standard criteria used by SR Research’s Eyelink systems to detect saccades. During this part of the task people are explicitly instructed to keep a steady fixation, thus we termed saccades during this period ‘intrusive saccades’ as opposed to the subsequent saccade made to the target after the fixation phase. Trials containing such 'intrusive saccades’ were subsequently removed from further analysis and these trials are thus not included in any of the other reported measures. Second, we determined the total displacement of the fixation position at a single point in time as the distance between the original fixation and the position of the eye at target onset in degrees. The total displacement measure is potentially more sensitive to slow one-directional drift as opposed to fixation area. The rationale behind this measure was that if an eye movement (below saccade thresholds described above) would go in a single direction then the overall fixation instability, as determined by the BCEA, would be relatively small since this measure is largely determined by the standard deviation of eye-movement in x and y directions. Thus the total displacement might reflect a different type of fixation instability. Third, fixation stability was calculated using the method of determining a bivariate contour ellipse area the methodology of which is extensive described elsewhere [23], [30], [34], [35].

To investigate the nature of the instability we analysed the power spectral density (using a fast fourier transformation) of the time-courses of the displacement [31], [41] of trials without saccades and blinks. The rationale behind this approach is that it might be more sensitive to detect a slow displacement drift as opposed to faster ‘jerky’ eye-movement instability that might be the result of reduced oculomotor control.

All measures were analysed using 2x3 mixed ANOVA with condition (1degree anchor vs. 3degree anchor) as within subjects factor and group (Control vs. MD vs. Simulation) as between subjects factor. Post-hoc t-tests (two-sided) were conducted using Bonferroni correction for multiple comparisons.

Apart from a diagnosis of JMD, inclusion criteria for the JMD group included a clear usage of a stable PRL as measured with the SLO and the ability to perform both a nine-grid calibration and validation on the Eyelink system prior to the start of the experiment. Four participants from the JMD group were unable to attend an SLO measurement and were thus excluded from the final analysed sample. In one case the PRL overlapped almost perfectly and was thus also excluded (MD6 in figure 1). If the number of valid trials, after removal of trials including an intrusive saccade, was more than 3 standard deviations away from the group mean, that subject was considered an extreme outlier. In the control and simulation group one extreme outlier on the intrusive saccade measure was excluded. In total our exclusion criteria thus led to a loss of 5 JMD participants, 1 control participant and 1 control participant performing the simulation experiment. The final analysed sample thus included 5 JMD participants (see Table 1 for clinical characteristics of the JMD group) and 9 controls matched for age and 5 controls participating in the simulation experiment. This relatively small number of JMD participants is not uncommon in the literature [16], [24], [29], [42], [43], [44]. In addition all our results figures include individual data points showing that our results are consistent across patients and that the behaviour of nearly each patient deviates from the control group.

## Results

**Figure 3:**
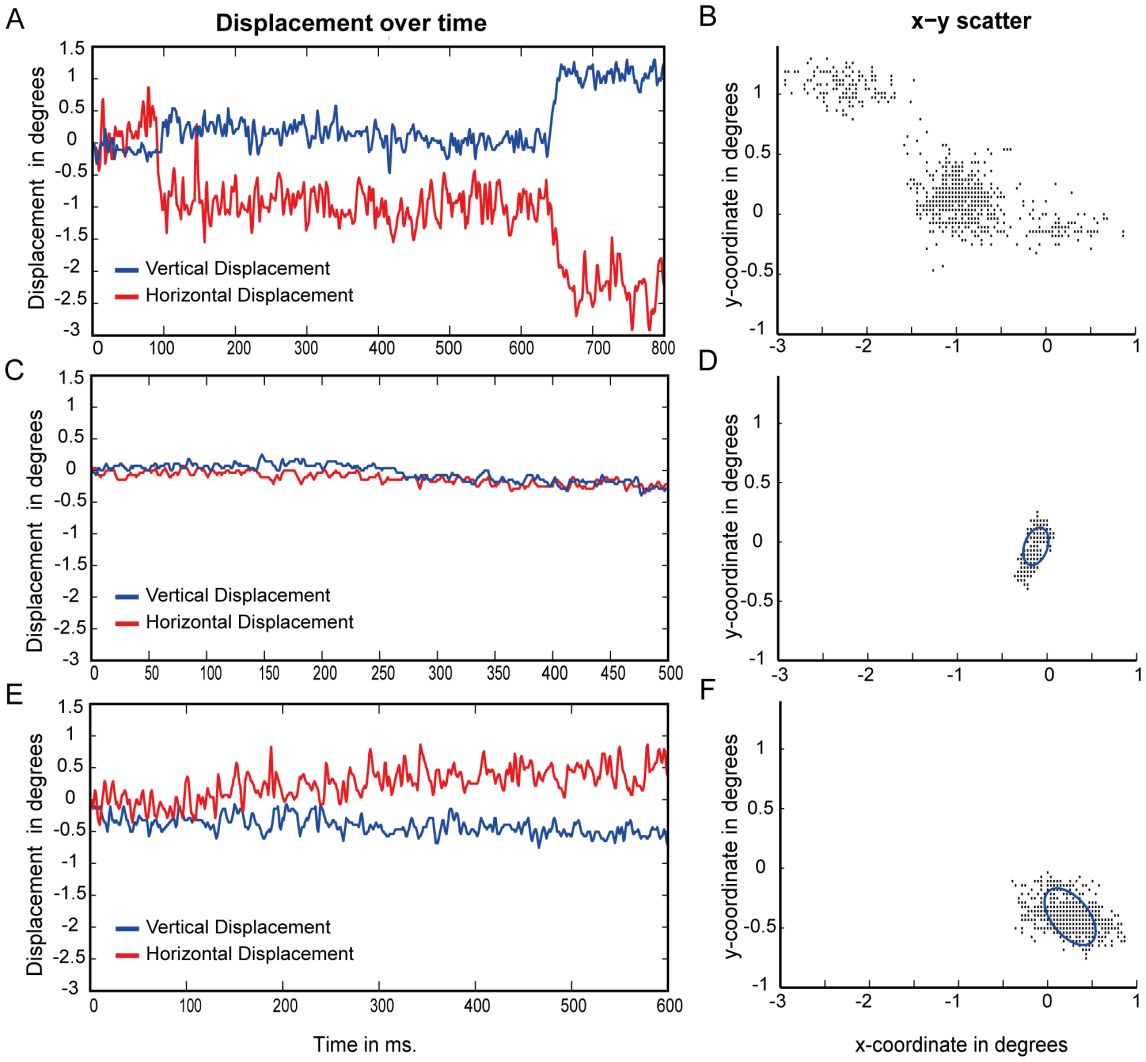
Time-course and scatterplot examples for different types of trials. Panels A, C and E show example time-courses of eye-position over time, lines indicate horizontal (red) and vertical (blue) displacement over time. The example time-courses illustrate measurement with (A) and without (C) an intrusive saccade in control subjects and a typical trial without saccades or blinks of a JMD patient (E). Panels B, D and F show the corresponding eye-positions across the entire measurement duration, indicating the effect of saccade and blinks on the displacement spread (Panel B). Displacement was summarized by the BCEA and examples are shown in blue circles in panels D and E. Only trials that did not include these intrusive saccades or blinks were included in the analysis of the BCEA, displacement and power spectral densities.

Example eye-movement recordings and BCEA computation are shown in Figure 3. Figure 3 depicts three types of trials. The top trial (3A and 3B) is an example of a trial with an intrusive saccade. The scatterplot (3B) shows how an intrusive saccade influences the spread of the displacement and thus the BCEA and overall displacement. Intrusive saccades can greatly bias the displacement and fixation stability measures and therefore all trials that included intrusive saccades were removed from the remaining analysis. The two other trials show a ‘normal’ time-course (3C) and scatterplot (3D) of a healthy control and a time-course (3E) and scatterplot (3F) of a patient with JMD. In the scatterplots of the included trial types (3D and 3F) examples of a BCEA are shown.

### Intrusive Saccades

**Figure 4:**
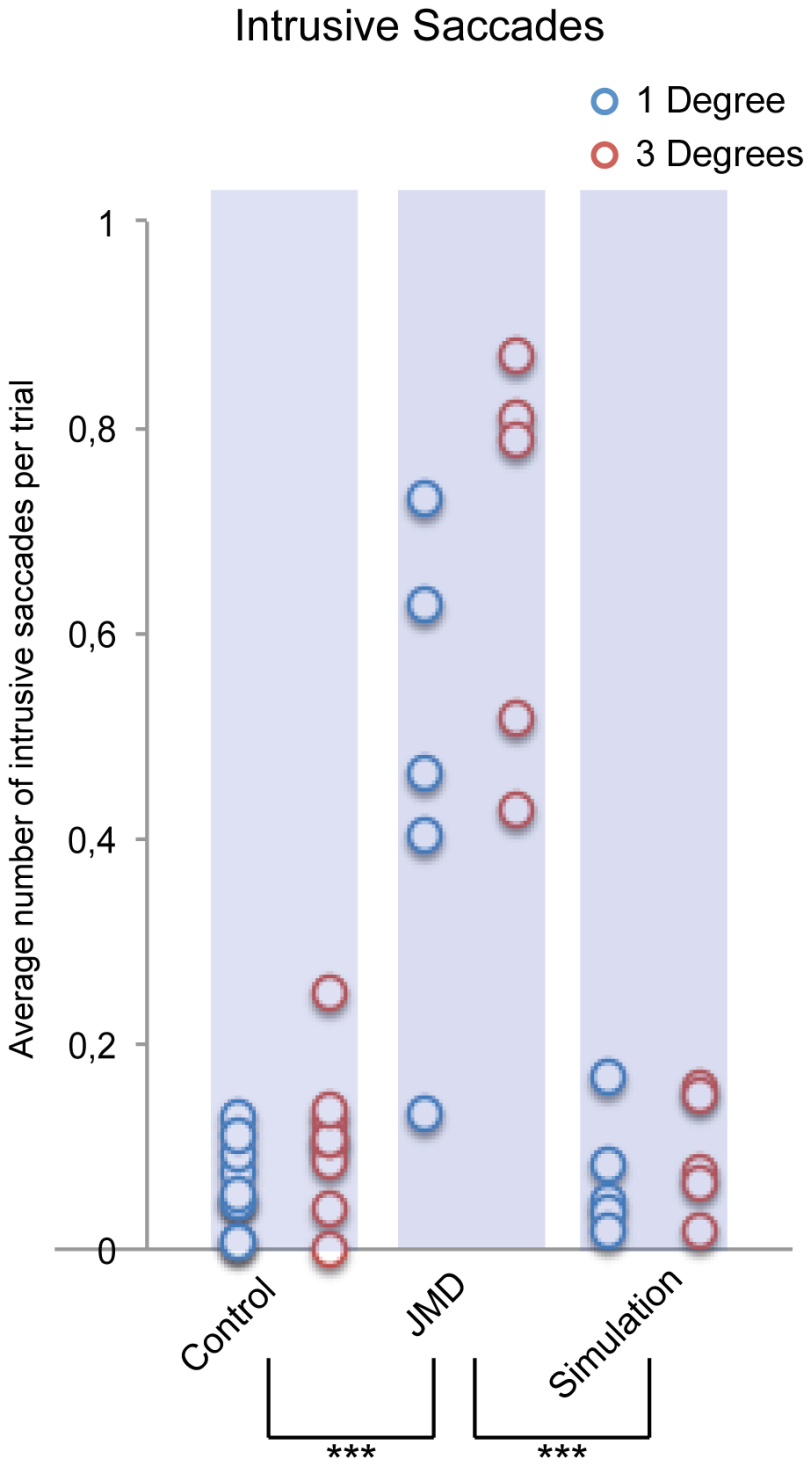
Number of intrusive saccades during fixation. The average number of intrusive saccades per trial are shown. Significant differences are marked: p<0.001 = ***, p<0.01 = ** & p<0.05 = *. This figure shows that the JMD group (1 degree [M: 0.47 SD: 0.23], 3 degrees [M: 0.68 SD: 0.20]) made significantly more intrusive saccades compared to healthy controls (1 degree [M: 0.06 SD: 0.04], 3 degrees [M: 0.11 SD: 0.07]) and controls performing the simulation (1 degree [M: 0.07 SD: 0.06], 3 degrees [M: 0.09 SD: 0.06]). It also shows that in the control group people made less intrusive saccade in the 1-degree condition.

We measured the amount of intrusive saccades, as defined above, during the fixation phase (figure 2A) of the fixation-offset paradigm. JMD patients made more intrusive saccades than both the control and simulation groups as is evident from a main effect of Group *F(2,16) = 46.243, p<0.001* and Post-hoc tests: JMD>Control (*p<0.001*) and JMD>Simulation (*p<0.001*), see figure 4. There was no apparent Post-hoc difference between the control group and the simulation group. These results clearly show that patients with JMD have difficulty in maintaining fixation even for a very short duration.

Our within subjects factor of fixation anchor-size also showed a main effect: F(2,16) = 8.746, p = 0.009. The interaction between the size of the fixation anchor and Group was not significant *F(2,16) = 3.373, p = 0.060*.

### Displacement

**Figure 5:**
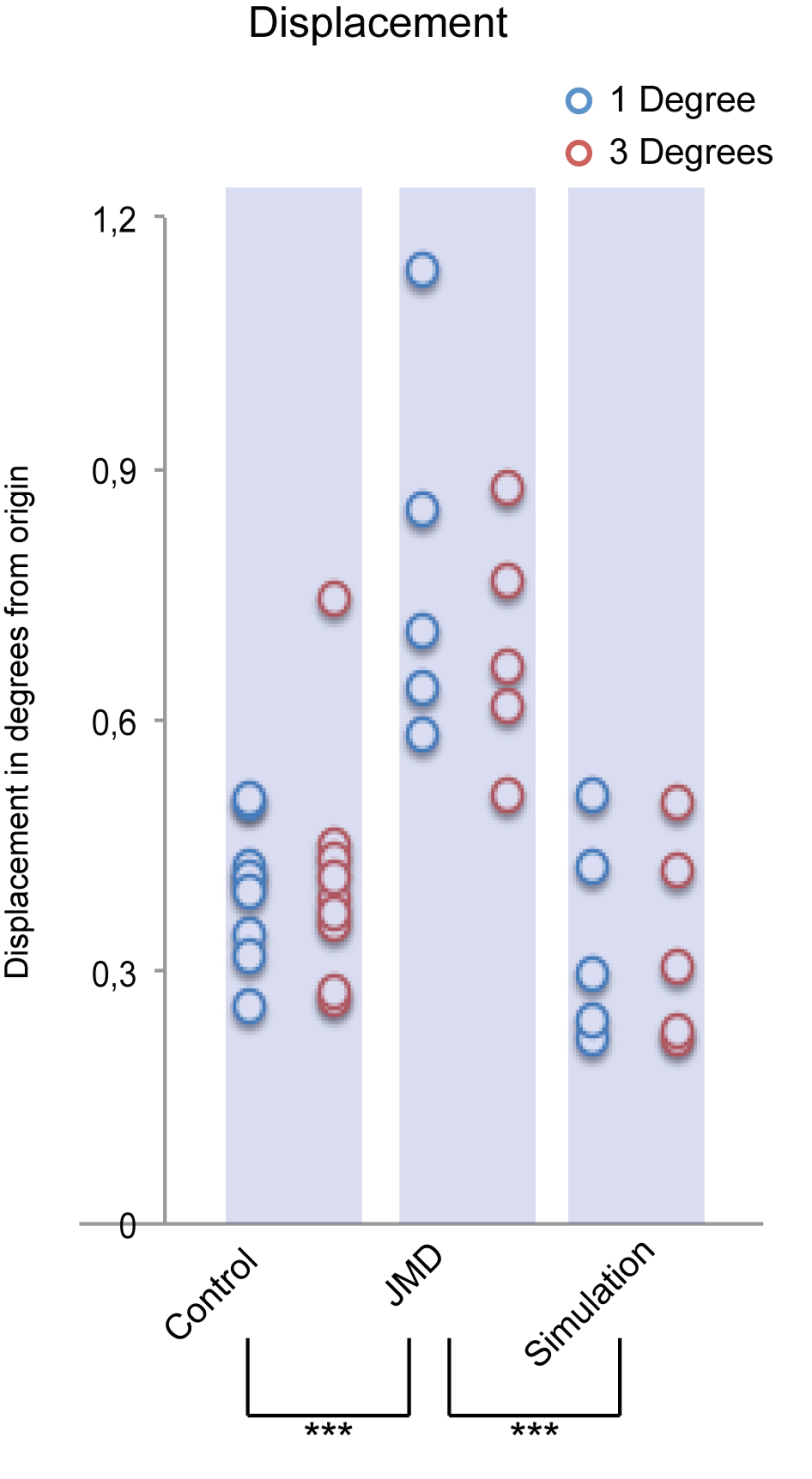
Displacement during fixation. The start-to-end displacement in degrees of visual angle is shown. Significant differences are marked: p<0.001 = ***, p<0.01 = ** & p<0.05 = *. This figure shows that patients with JMD (1 degree [M: 0.78 SD: 0.22], 3 degrees [M: 0.69 SD: 0.14]) deviated more from their original fixation at the end of the fixation phase compared to controls in both the normal (1 degree [M: 0.40 SD: 0.08], 3 degrees [M: 0.41 SD: 0.14]) and simulation (1 degree [M: 0.34 SD: 0.12], 3 degrees [M: 0.34 SD: 0.12]) paradigm.

Second, we measured the displacement between the start- and end-point of the eye at the onset of the fixation phase. Here, results show a main effect of Group *F(2,16) = 16.904, p<0.001*. This effect also seems to be mainly driven by the JMD group as Post-Hoc tests show: JMD>Control (*p<0.001*) and JMD>Simulation (*p<0.001*), see figure 5. There was no main effect of Anchorsize, nor an interaction effect on the total displacement. These results confirm that patients with JMD have an unstable fixation pattern even when trials that contained saccades were removed from the analysis.

### Fixation Stability

**Figure 6:**
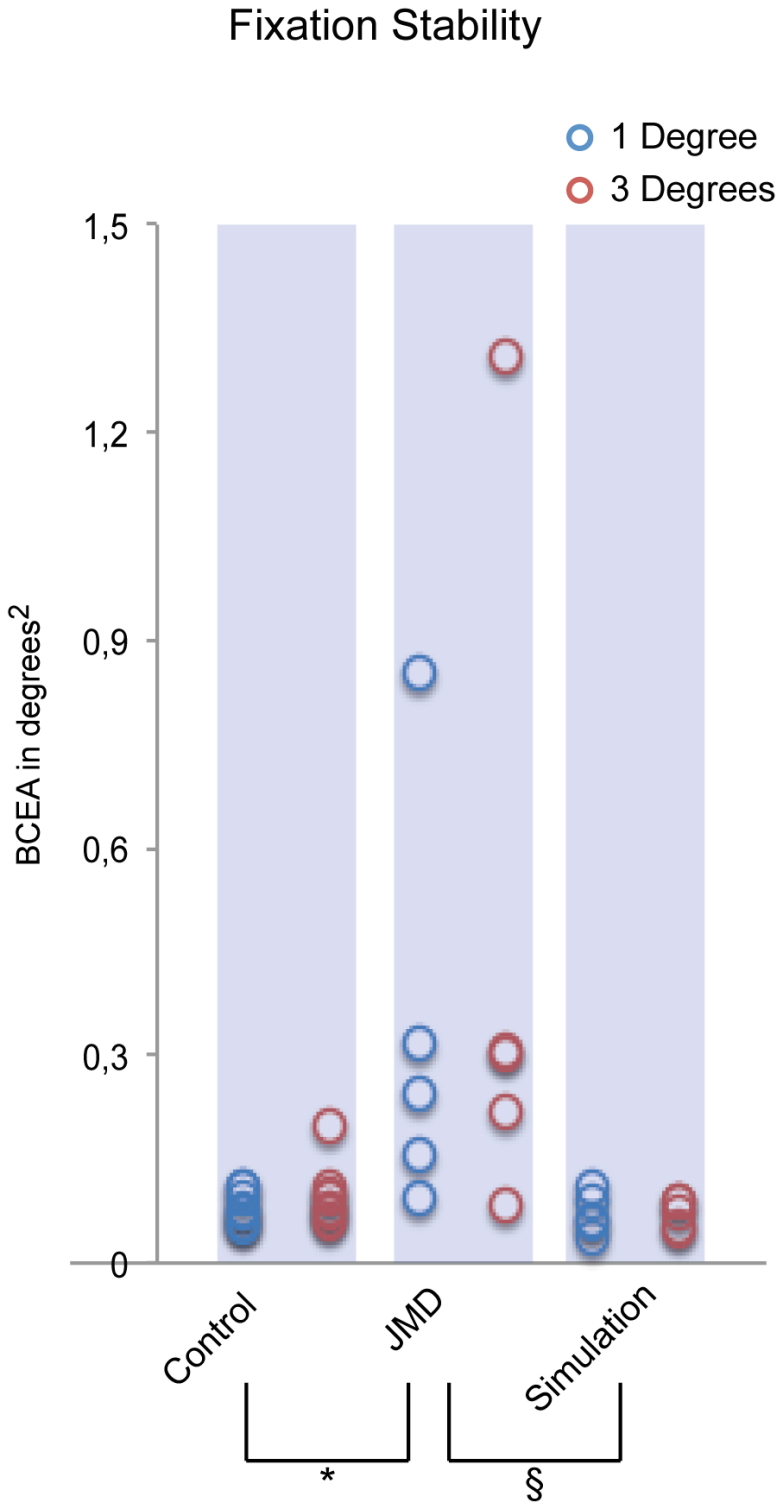
Fixation stability during fixation. The fixation stability in squared degrees as measured with a bivariate contour ellipse area (see figures 3B and 3D for an example). Significant differences are marked: p<0.001 = ***, p<0.01 = ** & p<0.05 = *. The “§” marks a trend. This figure shows that fixation was more unstable in the JMD group (1 degree [M: 0.33 SD: 0.20], 3 degrees [M: 0.44 SD: 0.49]) compared to the healthy control group (1 degree [M: 0.08 SD: 0.02], 3 degrees [M: 0.09 SD: 0.04]). The difference between the JMD group and the simulation group (1 degree [M: 0.07 SD: 0.03], 3 degrees [M: 0.07 SD: 0.02]) was not significant but a Bonferroni corrected p-value of 0.067 might be considered a trend.

Third, we determined the total fixation area using a BCEA for all time-points during the fixation-phase of the fixation-offset paradigm (Figure 3D & F). The results show that fixation stability as measured with a bivariate contour ellipse during a short fixation phase was significantly different across the three groups *F(2,16) = 4.476, p = 0.029*, see figure 6. Post-hoc tests show that this effect is mainly caused by the JMD group: JMD>Control (*p = 0.047*) and that there was no significant effect for the JMD group compared to the simulation JMD>Simulation (*p = 0.059*). There was no main effect of Anchorsize, nor an interaction effect on the fixation stability. The results remained statistically significant even with the JMD outlier removed (main effect of Group *(F2,15) = 11.537, p = 0.001* and Post-Hoc differences: JMD>Control, *p = 0.003* and JMD>Simulation, *p = 0.001*). Again, these results show that patients with JMD have an unstable fixation that cannot directly be related to an unnatural eye-position.

### Power Spectral Density

**Figure 7:**
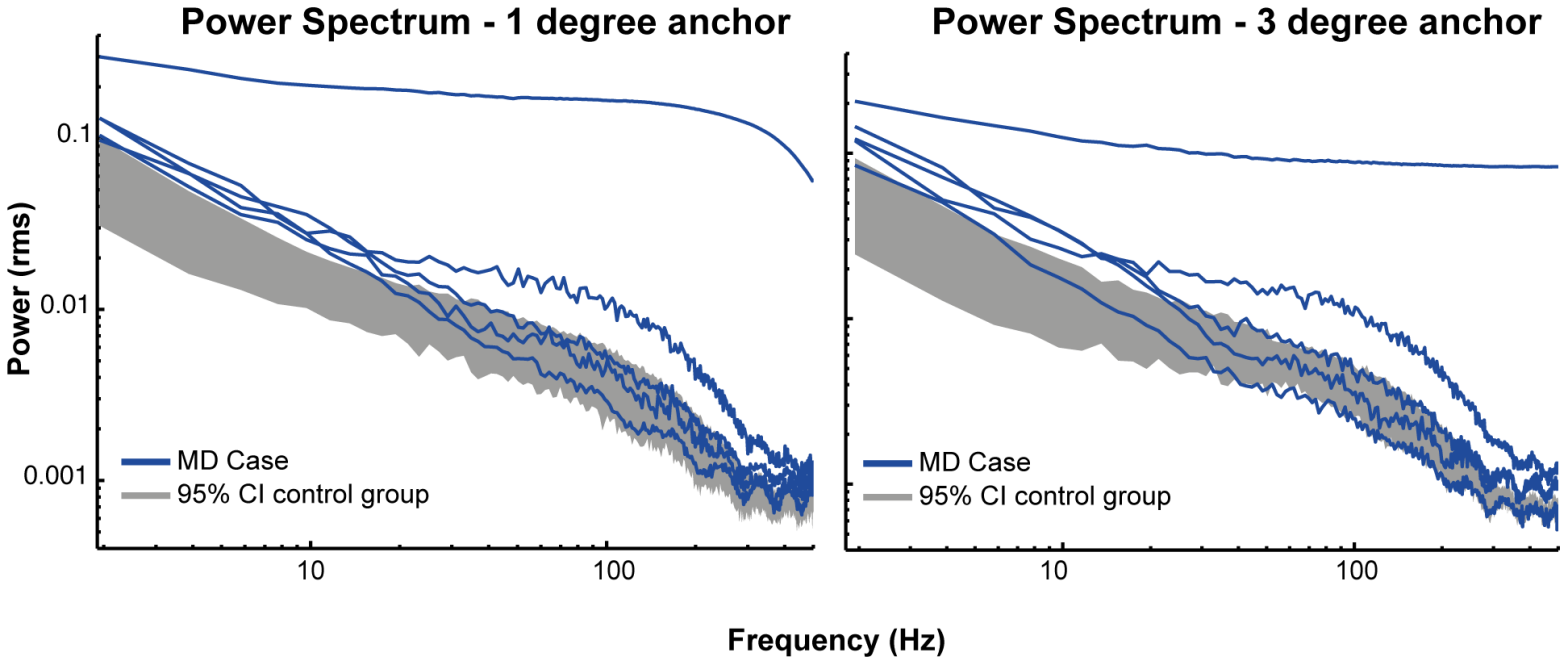
Power spectral density plots. Power spectral densities for the one and three degrees condition. The grey bars represent the 95% confidence interval (CI) of the control group mean. Each blue line represents one JMD patient. This figure shows that JMD patients had significant more power in the lower frequencies, indicating that low frequency eye-movements dominate the differences in displacement and fixation stability.

In order to measure the nature of the fixation instability we measured the power spectral density of the first 500 ms. of the fixation time-courses [31], [41]. The results from our power spectral density analyses are plotted in figure 7. This analysis suggests that the difference in fixation stability reported above is primarily driven by low frequency eye-movements. Figure 7 shows that below 10 Hz all patients from the JMD group fall well outside the 95% confidence interval of the healthy control group. Although the saccade parser used in the present study did not explicitly detect micro-saccades, low-frequency eye movements are often interpreted as slow variations in eye position such as drift [31], [45]. These low frequency differences can thus not be attributed to potential contamination with high-frequency eye movements such as micro-saccades or tremors [46].

## Discussion

The PRL in individuals with JMD exhibits unstable fixation patterns compared to fixation patterns of the healthy fovea. This instability is reflected in more intrusive saccades and decreased fixation stability. The decreased fixation stability is driven by slow variations in eye-position. The intrusive saccades measure we used in the present study might also reflect the fact that a decrease in visual acuity causes a deficit in terms of maintaining attention on the fixation stimulus. The decreased visual acuity might lead to uncertainty concerning the possible offset of the fixation cross, resulting in an increase in preliminary saccades away from the fixation stimulus, even though it is still physically present on the screen. Since all trials that contained such saccades were subsequently removed from further analysis this would not directly explain differences in fixation stability. Another potential cause for the detection of these saccades might have been a switch to a different PRL. Such a switch might be detected by the eye tracker as a rapid saccade, although it is unlikely that PRL switch will occur in such a short time-frame. If participants indeed switched to a different PRL the eye-tracker would have to be re-calibrated. Because it was calibrated for another PRL the drift check preceding each trial would fail after a PRL switch and would only continue after re-calibrating for the new PRL. Since no re-calibration was needed we assume that the final analysed trials were all from the same PRL.

The simulation suggest that it is unlikely that the present results can be explained by the notion that during peripheral viewing the eye-muscles are in an unnatural position and are thus more prone to saccadic behavior back to a more natural position. The control group performing the simulation shows similar fixation characteristics as the group performing the normal paradigm. Consequently, they also show the same differences when compared with the JMD group performing the normal paradigm. This is in line with previous research using simulated scotomas that show intact oculomotor control during reading [47] as well as during visual search [48]. Thus it is unlikely that any differences in oculomotor control in the patient group can be ascribed to peripheral vision alone.

Recently, there is a debate about the degree of plasticity and stability in the human brain following retinal degeneration. This debate is centred on the observation that regions deprived of retinal input, such as the foveal projection zone, can still respond to visual stimulation. Some authors have argued that these signals reflect cortical reorganization [42], [43], [49] whereas others don’t [29], [44], [50]. In the present study we found that in the control group fixation is more stable during presentation of foveal anchors (1 degree) compared to parafoveal anchors (3 degrees) as measured by the number of intrusive saccades. This increased stability was absent in both the JMD and the simulation group. Furthermore, the present results also show that peripheral fixation using a PRL is significantly more unstable in JMD patients compared to the simulation group. This indicates that the instability cannot be attributed to peripheral fixation alone. Thus even in the periphery where the PRL is located patients with JMD suffer from reduced fixation stability. This is in contrast to a previous study by White and Bedell [5] showing relatively stable fixation patterns in patients with bilateral macular disease. This study however used a much lower temporal resolution and might have been unable to accurately filter out intrusive saccades nor detect slow drift. Our results align with a more recent study that also showed decreased fixation stability in patients with JMD [51]. Given results from our previous study [29] it is likely that diminished visual acuity in the periphery contributes to this decreased fixational control. Although acuity was not measured in the present study, we assume that acuity diminishes at increased eccentricity for both controls and patients. With regards to plasticity, the behavioural eye-movement characteristics in the present study do not mimic the characteristics of the fovea when compared in a fixation-offset paradigm with foveal and parafoveal anchors. Therefore, the present results can be explained without the need for plasticity and instead be attributed to eccentric viewing with possible additional reduced visual acuity. We note, however, that current results might not extent to the non-dominant eye, as we only measured the oculomotor characteristics of the dominant eye Previous studies that focussed on AMD have shown that oculomotor control can differ between monocular and binocular viewing [21], [26]. Interestingly and in contrast to the present study, in AMD oculomotor control seems to be relatively good during binocular viewing [21]. As stated before it is possible that the relative late onset of AMD might allow for some sustaining of oculomotor control. In the present study we did however not test the effects of monocular versus binocular viewing. Future studies investigating oculomotor control of the PRL in individuals with macular degeneration should therefore carefully control for acuity and test both eyes separately to make such a direct comparison possible.

The differences in low-frequency eye-movements, interpreted as drift, provide another clue about the effect of macular degeneration on fixation characteristics. Under normal conditions drift is sometimes termed ‘slow control’ [52]. This refers to the balance between maintaining fixation on a certain object while allowing some eye-movements oscillation to prevent perceptual fading [13], [45]. As such, it is possible that the increased power of these types of eye-oscillations in JMD reflects a compensatory mechanism for loss of visual acuity due to peripheral viewing. It is also possible that this increased drift is simply a result of decreased oculomotor control as drift can sometimes also be triggered by spontaneous activation of peripheral oculomotor mechanisms [45]. Because we also observed an increased number of intrusive saccades in the JMD group the latter explanation seems the most likely. This finding is further supported by early findings showing that especially slow eye-movements are normally controlled by the fovea [53], although this study investigated fixation during a fairly long interval of 12 seconds and with considerably lower temporal resolution (<100 Hz).

In sum, our findings show a clear deficit in oculomotor control in patients with JMD during fixation. Given that we ruled out an unnatural eye-position as the cause for this instability we suggest that diminished peripheral acuity may be the most likely explanation. This may also be the factor underlying increased low-frequency drift. Perhaps, the adoption of a fixation stimulus that is scaled, such that is easier to see for the patients with JMD, might restore normal fixation behaviour.
